# Dry Matter Losses and Greenhouse Gas Emissions From Outside Storage of Short Rotation Coppice Willow Chip

**DOI:** 10.1007/s12155-015-9686-y

**Published:** 2015-10-29

**Authors:** Carly Whittaker, Nicola E. Yates, Stephen J. Powers, Tom Misselbrook, Ian Shield

**Affiliations:** Department of Agro-Ecology, Rothamsted Research, Harpenden, Hertfordshire AL5 2JQ UK; Department of Computational and Systems Biology, Rothamsted Research, Harpenden, Hertfordshire AL5 2JQ UK; Department of Sustainable Soils and Grassland Systems, Rothamsted Research, North Wyke, Okehampton, Devon EX20 2SB UK

**Keywords:** Biomass storage, Wood chips, Short rotation coppice willow, Dry matter losses

## Abstract

This study examined the dry matter losses and the greenhouse gas (GHG) concentrations within two short rotation coppice (SRC) willow wood chip storage heaps. One heap was built on a grassland area (East Midlands) and the other (Rothamsted) on a concrete hard standing. A series of 1- and 3-m probes were embedded in the heaps in order to retrieve gas samples for analysis, and pre-weighed net bags were positioned in the core of the heap to detect dry matter losses. The bagged samples showed dry matter losses of 18 and 19 % in the East Midlands and Rothamsted heaps after 210 and 97 days storage, respectively. The Rothamsted heap showed a whole-heap dry matter loss of 21 %. During this time, the wood chips dried from 54 to 39 % moisture content in the East Midlands heap and 50 to 43 % at Rothamsted. The results from analysing the whole Rothamsted heap indicated an overall loss of 1.5 GJ per tonne stored, although measurements from bagged samples in the core suggested that the chips dried sufficiently to have a minimal energy loss from storage. The process of mixing the heap, however, led to incorporation of wet outer layers and hence the average moisture content was higher in an average sample of chip. After establishment of the heaps, the temperature rose rapidly and this correlated with a peak in carbon dioxide (CO_2_) concentration within the heap. A peak in methane (CH_4_) concentration was also detected in both heaps, though more noticeably in the East Midlands heap after around 55 days. In both instances, the peak CH_4_ concentration occurred as CO_2_ concentrations dropped, suggesting that after an active period of aerobic decomposition in the first 2 months of storage, the conditions in the heap became anaerobic. The results from this study suggest that outside wood chip storage is not an efficient method of storing biomass, though this may be location-specific as there are some studies showing lower dry matter losses. It is necessary to explore other methods of harvesting SRC to minimise losses and optimise land use efficiency. Further research is required to detect whether there are fugitive emissions of CH_4_ from wood chip heaps, as this will compromise the net GHG savings from utilising the biomass stored in this way.

**Electronic supplementary material:**

The online version of this article (doi:10.1007/s12155-015-9686-y) contains supplementary material, which is available to authorized users.

## Introduction

Biomass is considered to play an important long-term role in reducing future global greenhouse gas (GHG) emissions, and since the implementation of the Renewable Energy Directive (RED) in 2009, there has been growth in the uptake of renewable energy in Europe [1]. Solid biomass, particularly wood and wood waste, is currently the biggest source of renewable energy in the EU and is expected to make a key contribution to meeting the EU’s 20 % renewable energy target by 2020 [2]. By 2020, 19.3 million ha of agricultural land could be diverted to dedicated bioenergy production, providing 100 Mtoe, while complying with good agricultural practice and without affecting domestic food production [3, 4]. Additionally, 40 Mtoe of forest biomass are envisaged to be available to biomass energy systems by 2020 without compromising environmental criteria [4].

In wood chip biomass supply chains, trees are harvested at a moisture content of around 50 % [5], and although it is possible to utilise wood chips with up to 65 % moisture content in modified furnaces or gasifiers, it is beneficial to dry the material to increase its net calorific value [6] and to reduce the quantity of water transported in the biomass [7]. The conventional harvesting window of willow is in the early spring; therefore, during this time large quantities of biomass are cut, which must be dried and stored until required. Forced-drying is one option but can be a costly process and requires access to facilities. A low-cost solution is to pile the wood chips into heaps. These can be placed under cover in open barns, or under tarpaulin or fleece sheets, though uncovered outside storage is cheaper and also benefits from direct sunlight and aeration in favourable drying conditions during April and May so that the chips can dry by natural ventilation [8]. Heaped storage also assists the logistics of the supply chain by buffering supply and enabling the movement of wood on demand [9] so that, for example, the discrepancies between the supply window and heat demand during winter can be addressed [5].

The process of piling wood chips in heaps is believed to lead to a redistribution of the moisture within the biomass, resulting in a wet outer surface and much drier inner part [9]. Open air heaps are often built to reach considerable dimensions in order to optimise the core to surface ratio [6, 10]. After 3 months of storage, a crust develops on the external surface which is believed to protect the remainder of the heap by allowing rainwater to run off [11]. The central region of the heap is then allowed to dry from 50 % moisture content in the spring to approximately 25 % by August [5]. The relationship between moisture content and drying time is assumed to be linear until this point [12]; however, there is evidence that weathering effects in the autumn can lead to a re-wetting of the chips to 45 % [5]. Therefore, the progression of drying can be affected by rainfall and relative humidity [13].

Wood is a biologically active material, and, unlike fossil fuel, it undergoes changes during storage [6]. Temperature in wood chip heaps typically rises rapidly as the material starts to decay [14]. Such a temperature change is a sign of microbial decomposition [5, 15], which can lead to material and energy losses [14]. As in composting, the process of degradation begins with the readily available nutrients that are released after the comminution process [16]. The breakdown of dry matter is then dependent on a number of factors including the age of material, the C/N ratio and moisture content of the material [14]. A suggested rule-of-thumb is that 1 % of dry matter is lost per month in outside chip storage [6], although total dry matter loss can be as high as 27 % for a 13-month storage process [17].

The breakdown process associated with increased heat in the heap can involve the rapid depletion of oxygen so that anaerobic conditions prevail in the core parts [18], which may lead to the generation of methane [19]. Little is known, however, about the extent to which this occurs [20]. There are concerns that dry matter losses and GHG emissions from wood chip storage have the potential to reduce the GHG-saving potential of biomass energy [19]. The goal of this study was therefore to investigate the dry matter losses and production of GHGs during storage of short rotation coppice (SRC) willow wood chips.

## Methods

### Wood Chip Heap Construction

Short rotation willow coppice plantations were harvested in spring 2014 at two sites in the UK. Both plantations were harvested using a Claas forager harvester with a Coppice Resources Ltd (Retford, UK) header.

#### East Midland Airport Site (Coordinates 52.835714, −1.327045)

The site consisted of two areas of SRC willow, one being planted in March 2010 (5 ha) and the other in March 2011 (6.75 ha). The areas were planted with a mixture of varieties consisting of Beagle (*Salix viminalis*), Resolution (*S. viminalis × Salix schwerinii*), Terra Nova (*S. viminalis* × *Salix triandra × Salix miyabeana*), Endeavour (*S. viminalis* “*Jorr*” *× S. schwerinii*) and Tordis (*S. viminalis × S. schwerinii*). Both sites were previously cropped in an arable rotation. The crops did not receive any fertiliser or pesticide applications during the growing period.

**Fig. 1:**
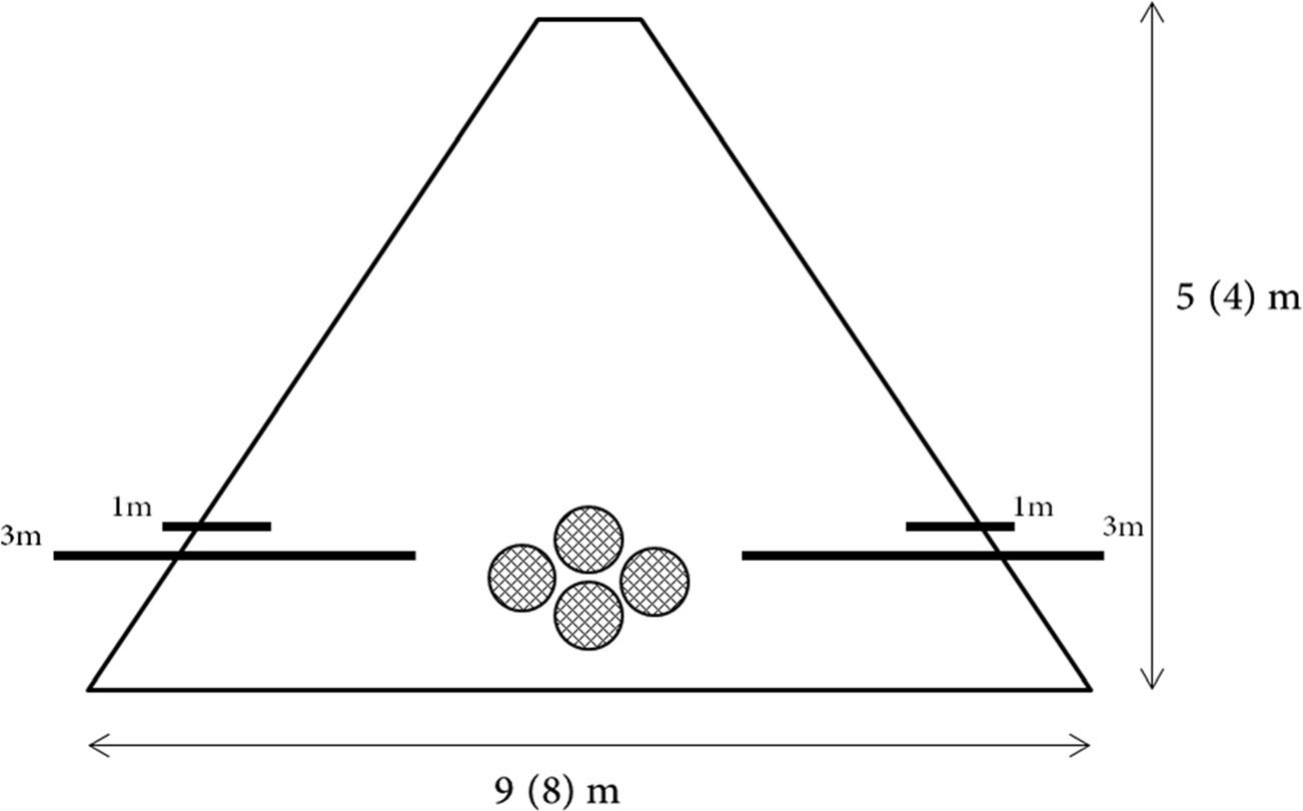
Diagram showing layout of GHG sampling probes and net bag samples. Dimensions of heap stated for East Midlands (Rothamsted heap in *brackets*). The East Midlands heap was 30 m long and the Rothamsted heap was 18 m long

The freshly harvested chip had a moisture content of 54 % (Table 1). The chips were tipped onto the ground and piled up using a tractor with a front mounted loader and bucket. During construction, some wood chips were spread on the ground to improve traction for the tractor. During heap build-up, the tractor drove over the bottom parts of the heap, compressing the material. The studied region of the heap was produced from the 2-year old crop which was harvested on 6 March 2014 and the heap was formed immediately after harvesting. The completed heap was approximately 30 m long (Fig. 1); however, the sampling area comprised the most southern 10 m end of the heap. The heap was estimated to consist of approximately 300 t of fresh material. The heap was built with the ridge following a rough east–west orientation, parallel to a predominant (80 %) westerly wind (P. Walker, personal communication).

**Table 1:** Average moisture, C, N and ash contents from different layers of the wood chip heaps from the Rothamsted and East Midlands heaps

Heap	Section/layer	Average content (%)				Number of samples
		Moisture	C	N	Ash	
East Midlands	Wood chips (initial)	54	48.2	0.4	1.1	8
	Outer layer (N)	27	49.0	0.5	0.1	5
	Outer layer (S)	10	49.0	0.4	0.9	5
	Wet layer (N)	50	49.5	0.7	2.6	2
	Mouldy layer	29	49.3	0.7	1.6	4
	Core	23	48.8	0.6	1.0	5
	Wet top section	71	49.1	0.6	1.3	2
	Mixed heap	39	48.8	0.6	0.9	5
	Net bag samples	31	49.0	0.5	0.9	11
	Ground sample	73	47.5	0.6	4.3	3
Rothamsted	Wood chips (initial)	50	48.3	0.3	1.6	9
	Outer layer (NE)	42	48.5	0.5	1.6	5
	Outer layer (SW)	44	48.7	0.4	1.6	5
	Mould layer	43	49.2	0.6	2.2	5
	Core	33	48.8	0.4	1.6	5
	Wet top section	68	48.9	0.5	1.3	3
	Mixed heap	43	48.8	0.4	1.6	5
	Net bag samples	29	48.4	0.4	1.7	19

#### Rothamsted Site (Coordinates 51.810306, −0.376591)

The site was planted in 2009 and consisted of varieties Endurance (*Salix dasyclados* × *Salix rehderiana*), Resolution (*S. viminalis* × *S. schwerinii*), Terra Nova (*S. viminalis* × *S. triandra × S. miyabeana*) and Tora (*S. viminalis* × *S. Schwerinii*). In contrast to the East Midlands site, herbicides and nitrogen fertiliser had been applied. The material for the heap was harvested on 3 April 2014. By this time, the crop was in leaf, with full bud flush (numerous, fully unfolded leaves observed) occurring between 18 March and 1 April (J. Cunniff, personal communication). The wood chip had a moisture content of 50 % (Table 1). The heap was built on a concrete standing using a similar method as for the East Midlands heap. Traction was not a problem here, and a telescopic loader was used so that very little of the chip was compressed. In this case, total heap mass was determined by weighing the wood chips during the heap establishment. The final heap was approximately 18 m long (Fig. 1). The heap was built with the ridge following a roughly north-west to south-east orientation. The wind direction is more variable here; however, the heap itself was sheltered somewhat by neighbouring buildings.

### Dry Matter Losses, Temperature and Quality Changes

To test for dry matter losses, at both sites, 20 random samples of approximately 3–4 kg of fresh harvested wood chips were collected from the tipped wood chip and placed into net bags. A temperature recorder (Log Tag® Model Trix-8, LogTag Recorders Ltd., Auckland, New Zealand) was added to each bag to record the temperature on an hourly basis throughout the storage period. The bags were tied, weighed and then returned to the wood chip heaps at approximately 1–2 m intervals across both faces of the heap, which were then buried during the piling up phase of the construction using the loader. Therefore, these bags were located in the core of the stack at least 2 m below the surface of the heap.

Nine random samples were taken of the fresh material. These were dried at 80 °C for 4 days to deduce the moisture content. Dried samples were ground using a hammer mill to pass through a 1-mm mesh. The ash component of the biomass was determined from the weight loss by initial drying at 80 °C for 12 h, and then in a muffle furnace at 450 °C for 4 h. The total carbon and nitrogen composition of the samples were determined using a LECO CN628 combustion analyser (LECO, Stockport, UK), based on a modified version of the Dumas digestion method.

### Greenhouse Gas Emission Sampling

During heap construction, gas sampling probes were horizontally inserted into the wood chip heap, following the methodology in Pier and Kelly [21]. In both heaps, ten 3 m and ten 1 m probes were used, inserted in pairs at 1–2 m intervals, and the vertical height of insertion recorded. The probes were split evenly between the two longest sides of the heap in order to increase the spatial resolution of the measurements [20]. The probes were constructed from stainless steel tubes with an external diameter of 21 mm and 2.65 mm wall thickness. The probe end inserted into the heap was pierced with 12 × 5 mm holes in order to facilitate gas diffusion into the probe. The external end of the probe was sealed with a cored rubber bung which held 8 mm gas impermeable plastic piping, to which a three-way polycarbonate Discofix stop valve was attached. All joints between the bung, steel pipe, plastic piping and tap were sealed with a neutral curing silicone (Dow Corning® 794 Glaze & Go, Dow Corning, Midland, USA). The taps were closed between samples. Gas sampling was initiated after 2 and 6 days at the Rothamsted and East Midlands sites, respectively. On each sampling occasion, a gas sample was taken from each probe and five ambient air samples were taken. Samples from the Rothamsted heap were taken at 2, 4, 6, 8, 11, 13, 15, 20, 28, 34, 40, 47, 55, 62, 71, 78 and 84 days after heap establishment and from the East Midlands heap at 6, 11, 13, 15, 18, 22, 27, 34, 41, 48, 55, 62, 69, 76, 83 and 112 days.

Gas samples were taken from the probes using a 60-ml syringe connected to the Discofix stop valve. After connecting the syringe and opening the valve, the syringe was emptied and filled three times to circulate the air within the probes before taking a 60-ml sample. Samples were then transferred to 22 ml pre-evacuated glass vials for transport and storage prior to gas concentration analysis. The gas samples were analysed for CO_2_, CH_4_ and N_2_O concentration using a Perkin Elmer Clarus 500 Gas Chromatograph (GC) linked to a Perkin Elmer TurboMartrix 110 headspace autosampler (Perkin Elmer, Waltham, MA, USA). The GC was fitted with a flame ionization detector (FID) housing a methanizer for measurement of CO_2_ and CH_4_ concentration, and an electron capture detector (ECD) for measurement of N_2_O concentration. Each gas sample was split between two identical Perkin Elmer megabore capillary Elite PLOT Q columns for delivery to the two detection systems. The FID was set at 350 °C whilst the ECD was set at 300 °C. A bracketed calibration employing five gas standards (mixtures of known amounts of CH_4_, CO_2_ and N_2_O in synthetic air) was used with each batch of samples, and check samples of known concentration were included at regular intervals within each sample run.

### Wood Chip Heap Breakdown

The East Midlands heap was dismantled after 210 days (1 October 2014). The Rothamsted pile was built on a working farm and that meant, for reasons of staff and machinery availability, that it had to be destroyed before the cereal harvests, after 97 days (9 July 2014). During dismantling, the piles were bisected using a tractor with a front-mounted loader so that the profile could be examined. Obvious layers were identified, the depth measured and five samples taken from each for analysis of moisture, nitrogen, carbon and ash content. The heaps were then broken down systematically using the front loader to retrieve the net bag samples. The bags were removed from the heap and reweighed, and data from the Log Tags® recorders were retrieved. Finally, the Rothamsted heap was re-weighed to enable total mass loss during storage to be calculated.

### Statistical Analysis

For each site, the method of residual maximum likelihood (REML) was used to fit a linear mixed model to each measured response (CO_2_, CH_4_ and N_2_O), consisting of random terms for the design used (probes and time points within probes) and fixed terms for the sampling zones to be tested (air vs. heap comparison, depth inserted, height from ground level and side of heap). The non-independence arising for the (non-equidistant) measurements over time within probes was accounted for by imposing a power model structure (a further random term) for time within probes, which naturally assumes that the correlation decreases as the time between measurements increases. The model was fitted assuming a common variance structure (same for all time points), and then with a different variance at each time point to test for heterogeneity of variance over time. Finally, spline terms in time were added to the model to test for significant curvature, either over time as a whole or separately for the different spatial locations (depth and side of heap) over time. Due to variance heterogeneity over time, data were analysed on the natural log scale for East Midlands, but for Rothamsted this transformation was only required for CO_2_. An adjustment of 0.1 was used for CH_4_ to account for some zero recordings under log-transformation.

Tests for random terms between possible models were based on change in deviance, distributed as Chi-squared on the change in degrees of freedom for any pair of nested, competing models. Data from the sites were not combined because there was only one replicate heap per site. The GenStat (16th edition, © VSN International Ltd, Hemel Hempstead, UK) statistical system was used for the analysis.

## Results

There was a change in colouration of the biomass, from green to brown, for both heaps during the course of the experiment. Also, there was extensive growth of *Agrocybe rivulosa* (wrinkled field cap) on the Rothamsted heap but not on the East Midlands heap.

**Fig. 2:**
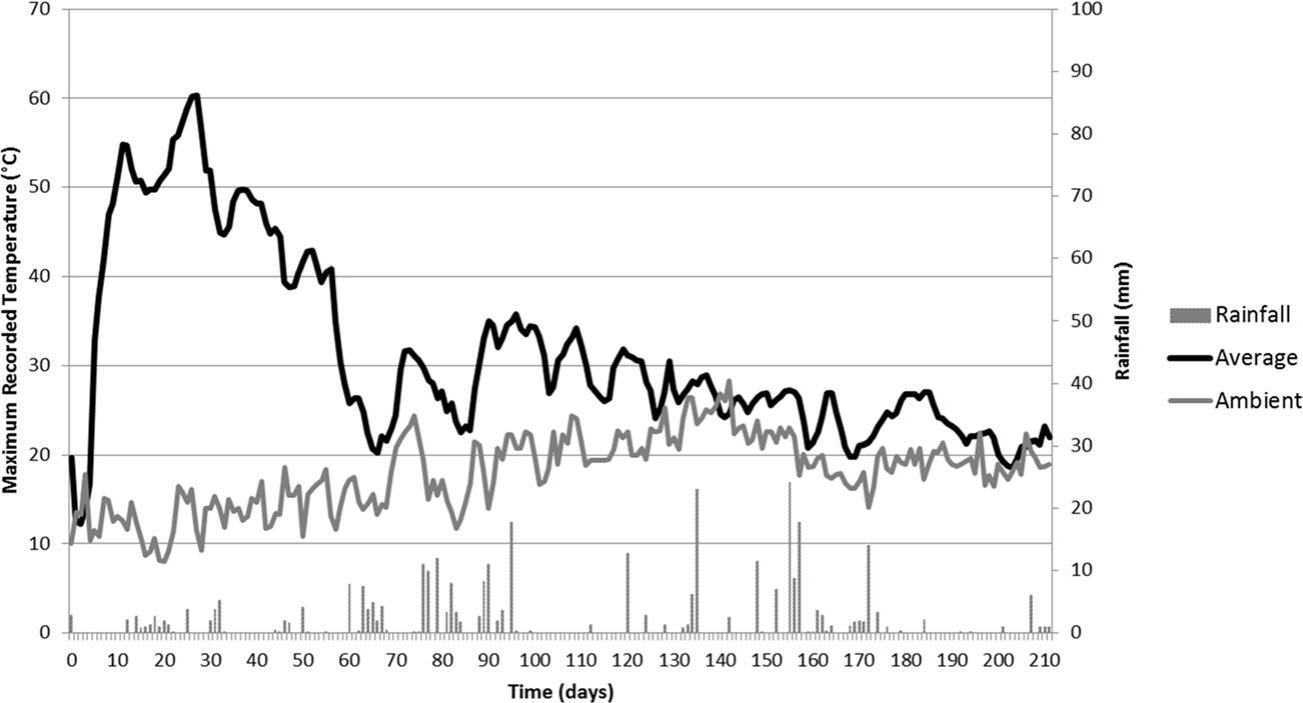
Average temperature records from net bag samples in the East Midlands wood chip heap, compared with ambient maximum temperatures. Day 0 = 6 March 2014

**Fig. 3:**
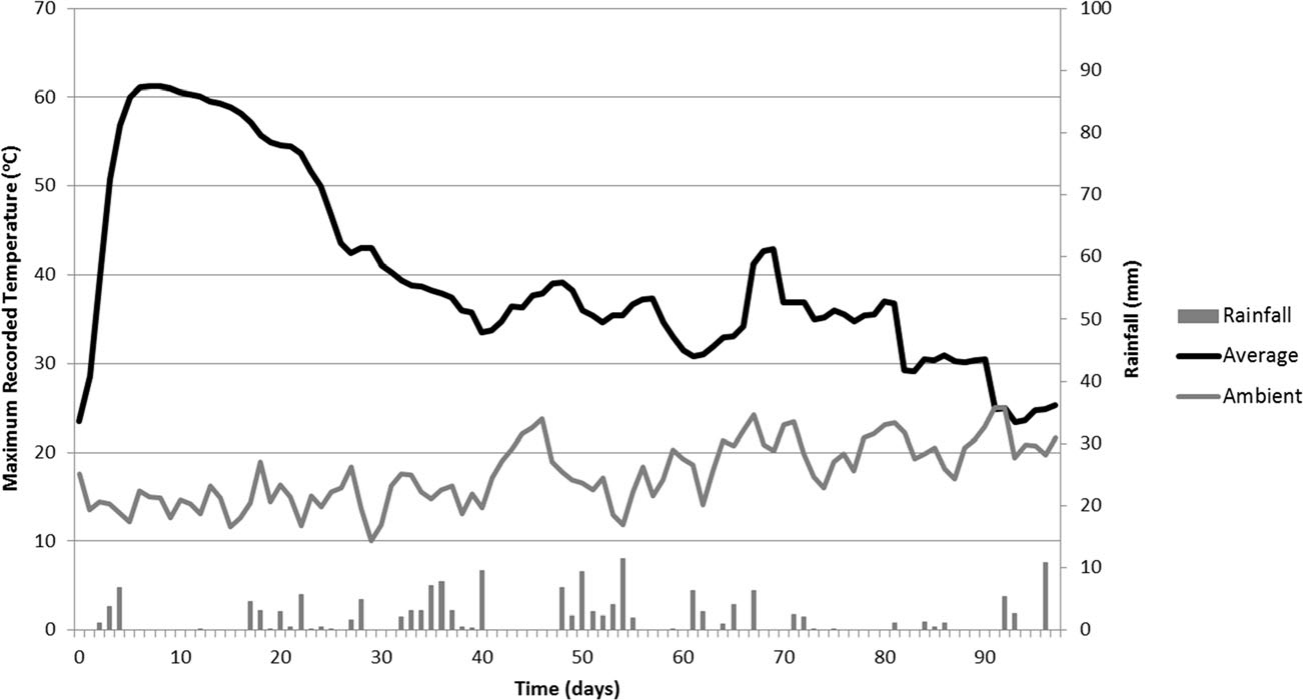
Average temperature changes over time from net bag samples in the Rothamsted wood chip heap, compared with ambient maximum temperatures. Day 0 = 3 April 2014

During the course of the experiment, a total of 334 and 164 mm of rainfall were recorded in the East Midlands and Rothamsted sites, respectively. Ambient temperature records and rainfall are shown in Figs. 2 and 3 along with the average (taken over the netted bag samples) internal temperature of the piles. Mean ambient temperatures were 18 and 16 °C, respectively.

Nine temperature recorders were retrieved from the East Midlands site. The records showed a rapid increase in temperature from 13 °C to a maximum of 66 °C after 12 days. Across Log Tags®, temperatures were recorded to be above 60 °C between day 3 and day 30. At day 27, the average temperature of the heap began to decline (Fig. 2). After day 56, there was a more rapid drop, after which heap temperature fluctuated between 20 and 35 °C.

All 20 recorders were retrieved from the Rothamsted pile. The maximum recorded temperature within the heap was 64 °C on day 6. Readings from the Log Tags® showed a consistent rapid increase in temperature from 24 °C to just over 60 °C over a period of 5 days (Fig. 3). A temperature of 60 °C was maintained for a period of 19–29 days, after which the temperature declined. Between day 31 and the end of the experiment, the temperature ranged between 30 and 40 °C. Comparing the two heaps, the Rothamsted one heated up more rapidly, reaching peak average temperature (61 °C) after 6 days of establishment compared to 27 days for the East Midlands heap (60 °C). Overall, the Rothamsted heap had 32 days over 40 °C compared to 46 days for the East Midlands heap. Temperature patterns fluctuated between 20 and 40 °C after day 65 at East Midlands and after day 45 at Rothamsted. It therefore appears that the temperature increase occurred faster at Rothamsted, where ambient temperature was less variable, and where there was a long period of dry weather for 12 days following 5 mm of rain on day 4.

### Heap Breakdown Observations

After bisecting the heaps, various layers were identified (Table 1). At the East Midlands site, four layers were identified: the outmost layer was approximately 10 cm deep and much drier on the southern (S)-facing side. Below this on the S-facing side there was a distinct white mould layer which ranged between 15 and 30 cm deep, under which the drier core region was found. On the northern (N) side, the layers were more heterogeneous with varying depths of mould, though sometimes it was absent, being replaced with a darker wet layer absent on the S-facing side. The central regions of the heap were generally dry. The Rothamsted heap was similar, except there was no darker layer present. The wood chips forming the apex of both heaps were extremely wet.

As the East Midlands heap had been positioned on grass, it was necessary to leave a layer of chip on the soil to eliminate contamination. Taking 21 samples along a transect bisecting the remaining bed found that this was on average 15 mm deep (95 % confidence interval (CI) 14–19), but ranged between 5 and 30 mm. Four samples taken from the bed averaged 63 kg fresh material (95 % CI 60–66) per m^2^, with a moisture content of 65 %, (95 % CI 63–66) resulting in a dry matter loss of 22 kg/m^2^, or approximately 6 tonnes for the whole heap. Measuring the additional loss from the use of wood chips to drive over the ground during heap construction was difficult, as compression from the vehicles meant that lower layers of the chips were fully embedded into the ground. However, the driving bed had an estimated footprint of 86 m^2^. Four random quadrat samples taken from the area weighed 22 kg/m^2^ (95 % CI 20–25) at 69 % moisture content (95 % CI 65–72) suggesting a loss of up to 1 t dry matter for this area in addition to losses under the heap. Analysis of this material showed the ash content to be 3.7 times higher than that of the original wood chip, which is most likely due to soil contamination. At the Rothamsted site, losses to the ground and surrounding area were negligible.

### Dry Matter Losses and Compositional Changes

At the East Midlands site, samples of the initial fresh chip had average moisture content 54 %, 95 % CI 52–54 %. Samples of the mixed heap showed that biomass had dried to 39 % (95 % CI 30–48 %) after 6 months (Table 1). Unfortunately, only 13 of the 20 bagged samples were found in the East Midland pile. Of these, two had been damaged and opened, therefore could not be used for dry matter (DM) loss assessment. The mean moisture content of the net bag samples from the core region of the heap was 31 % (95 % CI 21–42 %), although this was skewed somewhat by the two particularly wet and mouldy bags (62 and 78 % moisture content); without these, the average was 23 %, which was the same as the core. This indicates a successful rate of drying within the heap; however, after mixing the heap with the wetter outside layer, the average moisture content increased. The average dry matter loss of the netted bags was 18 % (95 % CI 13–23 %), with the highest loss (46 %) occurring in the 62 % moisture content bag. Without the two particularly wet bags, the average dry matter loss was 14 %.

At the Rothamsted site, 84,060 kg of freshly cut biomass was stored (Table 2). Samples showed the chips had an initial moisture content of 50 % (95 % CI 47–53 %) and had dried to 43 % after 4 months (95 % CI 32–54 %). Only 1 of the 20 bags was lost through damage. The average moisture content of the remaining net bags was 29 % (95 % CI 27–31 %), which is slightly drier than the sample for the core. Out of the 19 bags examined, 9 dried down to between 22 and 25 % moisture content, and the overall range was 22 to 34 %. The average dry matter loss of the netted bags was 19 % (95 % CI 17–20 %). The measured mass of the wood chip heap at the end of the experiment was 58,030 kg, corresponding to an estimated dry matter loss of 9062 kg or 21 % of the dry matter of the stored biomass.

**Table 2:** Summary of dry matter losses in the Rothamsted heap

Phase	Measurement	Value	Units
Start of experiment	Mass of chips	84,060	kg
	Moisture content	50	%
	Start dry matter	42,269	kg
After storage	Mass of chips	58,030	kg
	Moisture content	43	%
	Dry matter content	33,207	kg
	Loss	9062	kg

There was high variability in the changes in nitrogen and ash composition between the bags at the East Midlands site, with no statistical difference (*p* > 0.05, *t* test) between before and after storage (Table 1). At the Rothamsted site, the original wood chip had a significantly lower N content than the East Midlands heap (*t* = −4.34, d.f = 4, SED = 0.00054, *p* < 0.001). At the Rothamsted site, there was a significant increase in the percentage N content between the bagged samples and original chip (*t* = −1.99, d.f = 20, SED = 0.0017, *p* < 0.05), but a zero change in relative C, resulting in an decrease in the C/N ratio after storage. Compared to the bagged samples found within the heap, there were significant (*p* < 0.05, *t* tests) increases in C and N proportions in the outer layers, particularly the mouldy zone. There were no clear differences in ash contents of the Rothamsted wood chips before and after storage.

Using the information on moisture content and composition, the Milne equation and data in the Phyllis Database were used to predict the lower heating value (LHV) of the wood chips [22], using the average figure for willow (untreated wood, willow). The LHV was estimated at 7.2 and 9.9 GJ/t before and after storage at the East Midlands pile. When accounting for the dry matter losses, there was an average net energy loss of 1.1 GJ/t stored, as estimated from all the net bags. The highest losses (2.1 and 3.5 GJ/t stored) were recorded in the wettest bags (78 and 62 % moisture content bags, respectively). At the Rothamsted pile, the LHV of the mixed heap was estimated at 7.8 and 9.1 GJ/t before and after storage. Factoring in dry matter losses leaves a net energy loss of 129 GJ for the whole heap, or 1.5 GJ per t stored. The estimated energy losses from the average of all of net bag samples within the heap were small (0.1 GJ/tonne stored). This was due to the acceptable level of drying in the bags as they were situated in the core. After the heap was moved, re-tipped and mixed however, the outer layers and chimney zone meant that the average moisture content of a random mixed heap samples was 10–16 % higher than in the core and hence a net energy loss was calculated.

### GHG Concentrations in the Wood Chip Heap

#### East Midlands Heap

**Fig. 4:**
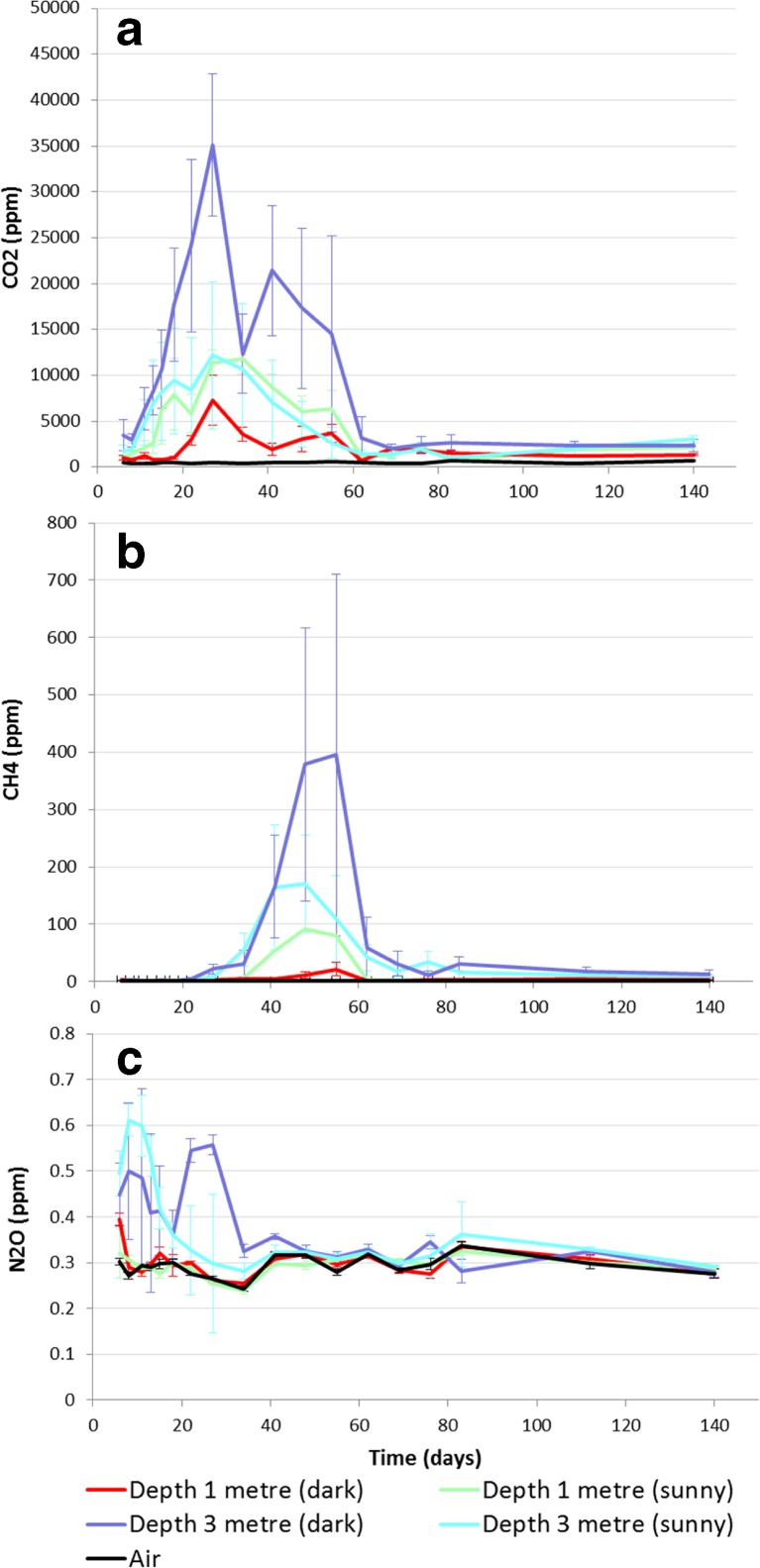
Average greenhouse gas detection from 1- and 3-m probes from the East Midlands wood chip heap (with standard error), showing CO_2_ (**a**), CH_4_ (**b**) and N_2_O (**c**). Day 0 = 6 March 2014

There was a rapid increase in CO_2_ concentrations in the first 30 days of the experiment, with the peak (35,000 ppm) occurring on day 27 on the darker, north side of the heap (Fig. 4a). On a number of occasions, the CO_2_ levels observed were off scale (FID attenuation settings selected were a compromise in order to detect peaks across a broad range of both CH_4_ and CO_2_ concentrations), with the truncated CO_2_ peaks thus providing an under-estimate of the actual concentration. After 60 days of sampling, the concentration levelled out at around 1500 ppm and remained roughly constant until sampling ceased. After the peak in CO_2_, a steady increase in CH_4_ concentrations was recorded (Fig. 4b), again on the darker side, with the maximum average concentration (275 ppm) occurring on day 48. The highest single probe measurement for methane was 1633 ppm, on day 55. By day 69, CH_4_ concentration had declined to an average of 13 ppm and remained roughly constant until the end of the experiment. Nitrous oxide concentrations were highest early in the experiment, with the greatest variation occurring at days 6 and 8. The peak average N_2_O concentration was 0.56 ppm, occurring on the south-east or ‘sunny’ side. There was a downward trend in the N_2_O concentrations from the probes at 3 m after the first sampling (Fig. 4c). Correlation of the average 3-m probe GHG concentrations against the average temperature of the heap showed a significant positive relationship for CO_2_ (*R*^2^ = 0.6, *n* = 18, *p* < 0.001) and N_2_O (*R*^2^ = 0.4, *n* = 18, *p* < 0.005) with temperature. There was no significant correlation between CH_4_ concentration and heap temperature (*p* = 0.800).

The linear mixed model fitted for log(CH_4_) and log(N_2_O) was:

$$ y= vHeight+\left( AirvsHeap/\left( Depth* Side\right)\right)* vDay+Spl\left( vDay+ vDay. Depth\right)+\left( Probe/ Day\right), $$

where *y* represents log(CH_4_) or log(N_2_O), the slash (/) indicates a nesting of model terms, a star (*) indicates the main effects and interactions between terms, and *Spl*(*vDay* + *vDay.Depth*) indicates the variance component of the curvature with respect to days modified with respect to depth. Here, *vHeight* and *vDay* are variables, and all the other terms are factors: *AirvsHeap* accounting for the control samples, *Depth* and *Side* accounting for these sample zone effects nested within the heap, and *Probe*/*Day* extracting variance due to the time points nested within probes. The model for log(CO_2_) was the same but with *Spl*(*vDay* + *vDay.Depth*) being replaced by *Spl*(*vDay* + *vDay.Side*), as curvature was different with respect to the two sides rather than between the two depths of the probes (Supplementary data).

**Fig. 5:**
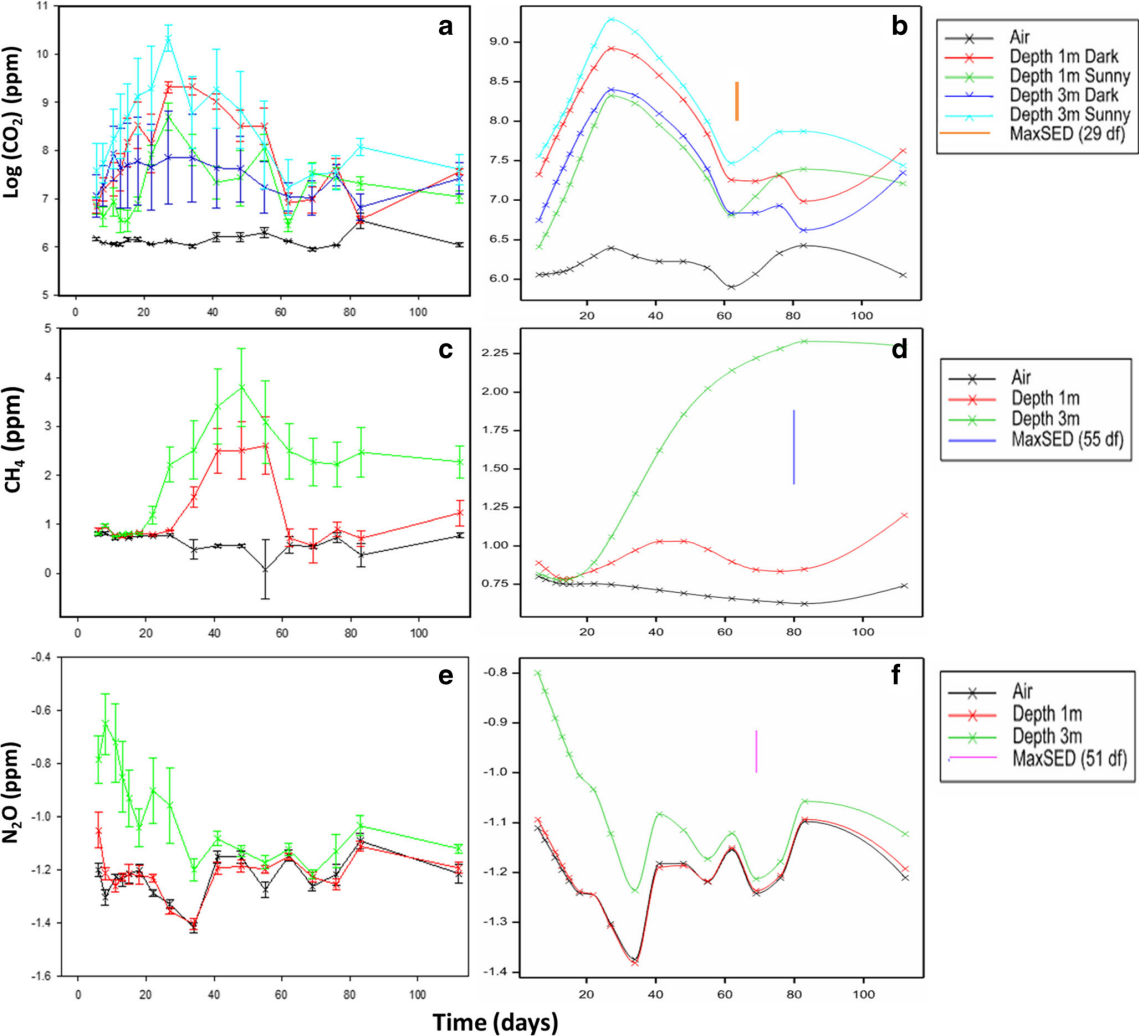
East Midlands heap predicted means (with standard errors) for GHGs CO_2_ (**a**), CH_4_ (**c**) and N_2_O (**e**) and trends for each GHG (**b**, **d**, **f**) recognised by the spline term with significant (*p* < 0.05, *F*-test) main effect (or interaction) terms for depth and/or side

Given the repeated measures from probes, concentrations of all three GHGs showed correlation over time that was heterogeneous, i.e. significantly different (*p* < 0.05, *χ*^2^ tests), over the time points (Supplementary data). Following this, the *F*-tests for the fixed effects indicated a significant interaction (*F* = 5.44 on 1 and 29 df, *p* = 0.027) between *AirvsHeap*, *Depth*, *Side* and *vDay*, for CO_2_ (Fig. 5a), and between *AirvsHeap*, *Depth* and *vDay* for CH_4_ (*F* = 20.15 on 1 and 55 df, *p* < 0.001) (Fig. 5c) and N_2_O (*F* = 14.86 on 1 and 44 df, *p* < 0.001) (Fig. 5e). There was therefore an effect of side for CO_2_ that was not seen in the other gases.

Figure 5 shows the trends for each GHG that are recognised by the spline term along with the significant (*p* < 0.05, *F*-test) main effect (or interaction) terms for depth and/or side. For all gases, the concentrations were noticeably higher from the deeper probes (Fig. 5b, d, f). For CO_2_, there was an opposite effect of depth and side: where the highest concentrations were from the 3-m probes of the sunny side of the pile but in the 1-m probes of the dark side. Hence, there was evidence of a different trend over time for the five sample zones (air, Dark 1 and 3 m, Sunny 1 and 3 m) albeit with the *shape* of the trend (in the spline term) being modified with respect to side. There was also an overall negative relationship of CO_2_ with height (*F* = 6.19 on 1 and 18 df; *p* = 0.017; coefficient −0.0083, SE 0.0027). This effect was not observed in the CH_4_ or N_2_O results.

#### Rothamsted Heap

**Fig. 6:**
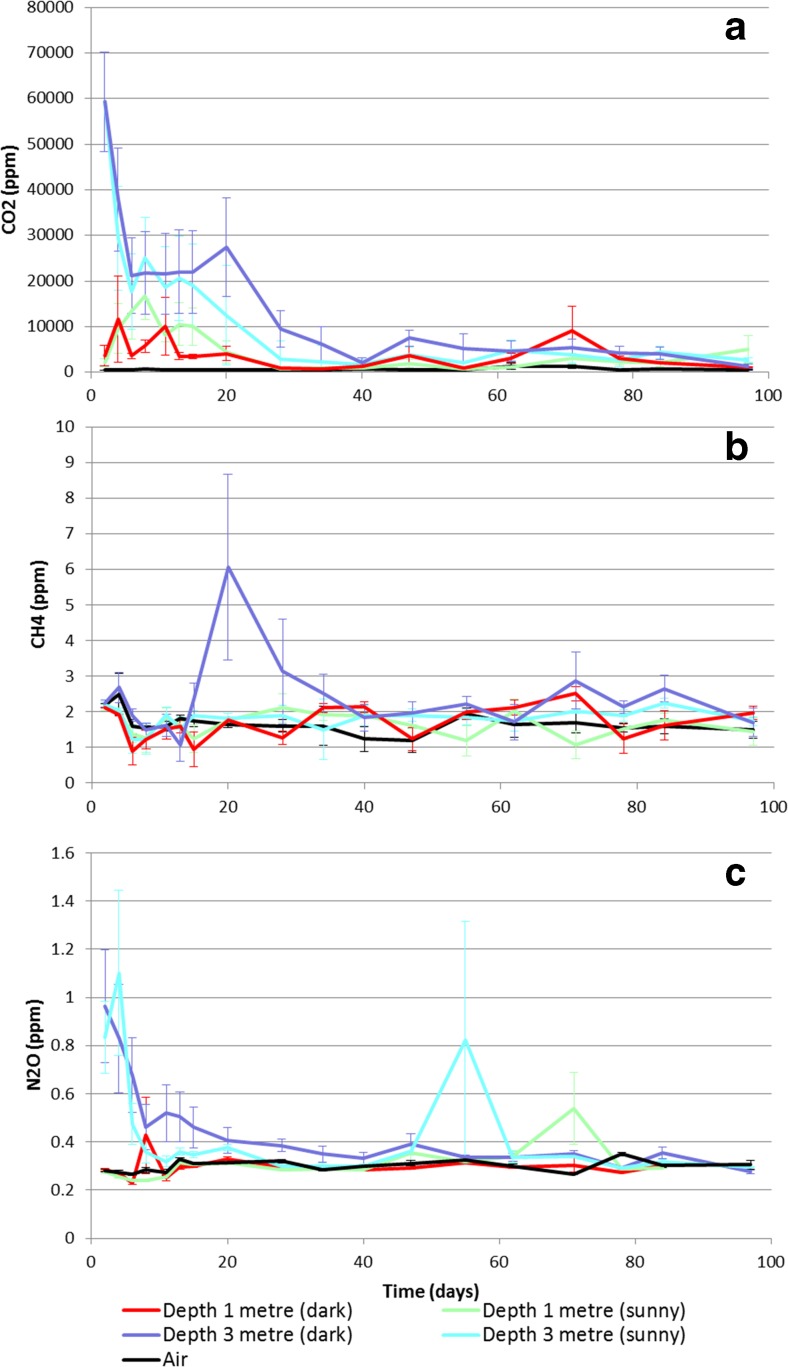
Average greenhouse gas detection from 1- and 3-m probes from the Rothamsted wood chip heap (with standard error), showing CO_2_ (**a**), CH_4_ (**b**) and N_2_O (**c**). Day 0 = 3 April 2014

The statistical analysis did not directly compare the two heaps because there was no replication of the heaps at the sites; these also being confounded with method of construction (on concrete or soil). A general inspection of the results shows that the CO_2_ concentrations in the Rothamsted heap were far higher in the first few days than those detected in the East Midlands heap; however, CO_2_ concentration fell more rapidly, following a peak at day 4, to a similar level as in the East Midlands heap, within a period of 34 days (Fig. 6a). The concentrations then dropped to a lower level (2500 ppm) that was near constant until the end of the experiment. Correlation of the average 3-m probe GHG concentrations with the average temperature of the heap gave a significant positive relationship (*R*^2^ = 0.26, *n* = 18, *p* < 0.05) for CO_2_. There were no such correlations for N_2_O or CH_4_ (*p* = 0.180 and 0.870, respectively); however, over the first 2 weeks only, across all probes, there was a significant negative relationship between N_2_O concentration and the heap temperature (*R*^2^ = 0.14, *n* = 60, *p* < 0.005). For CH_4_, the greatest variance occurred at days 20, 28 and 34, when there was an obvious, yet brief peak of CH_4_ detected for the 3-m probes on day 20 (Fig. 6b). After the peak, the CH_4_ concentrations did not stabilise in the same way as the CO_2_ concentrations did, but continued to fluctuate. As for CO_2_, the peak in CH_4_ concentration was earlier than seen in the East Midlands heap. Similar to the CO_2_, there was a very early peak in N_2_O concentrations in the Rothamsted heap (Fig. 6c), with the greatest variance at day 4. The peak was higher than that observed in the East Midlands heap (0.95 vs. 0.55 ppm).

**Fig. 7:**
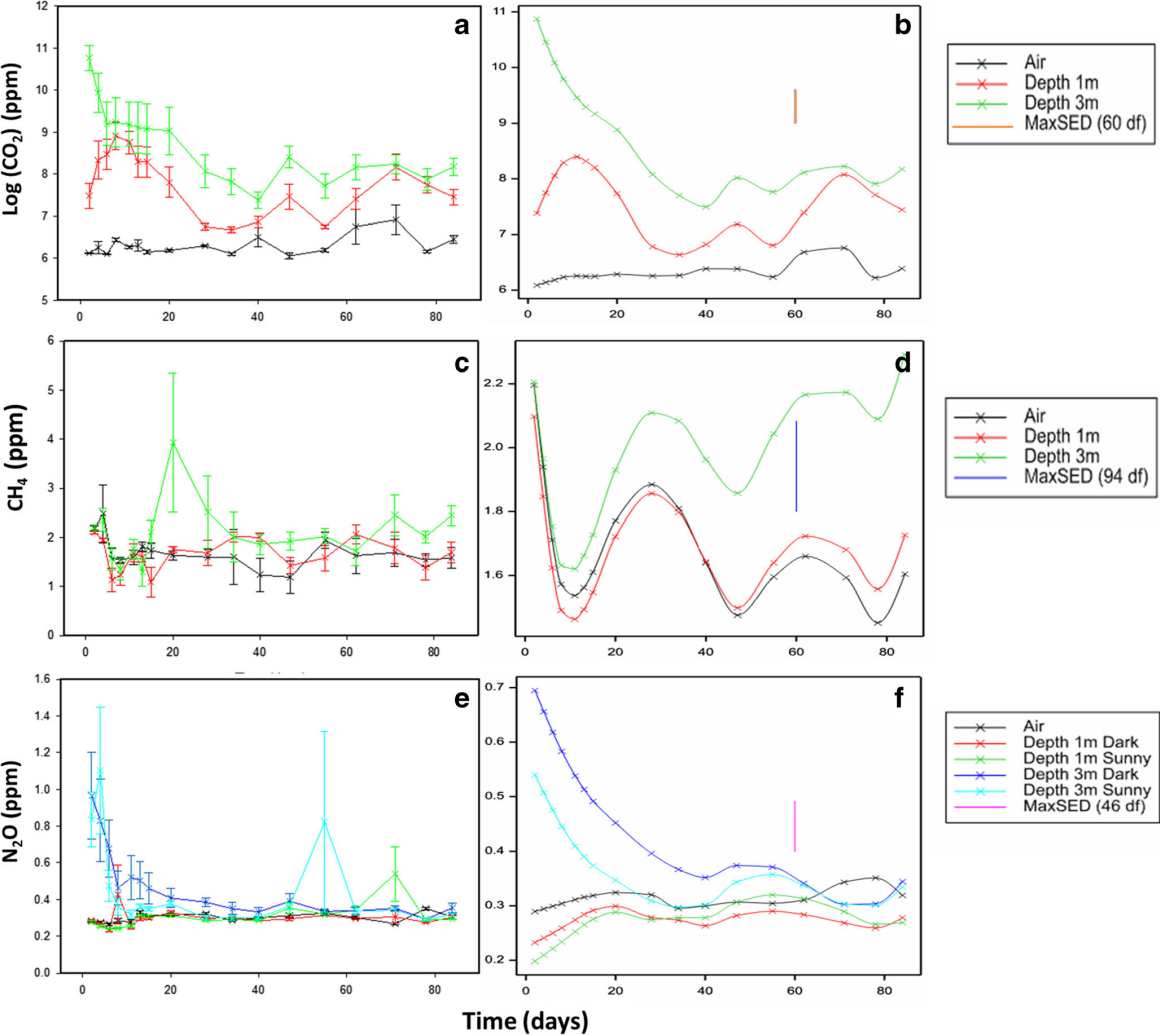
Rothamsted heap predicted means (with standard errors) for GHGs CO_2_ (**a**), CH_4_ (**c**) and N_2_O (**e**) and trends for each GHG (**b**, **d**, **f**) recognised by the spline term with significant (*p* < 0.05, *F*-test) main effect (or interaction) terms for depth and/or side

The linear mixed model fitted for log(CO_2_) was:

$$ y= vHeight+\left( AirvsHeap/\left( Depth* Side\right)\right)* vDay+Spl\left( vDay+ vDay. Depth\right)+\left( Probe/ Day\right), $$

with terms as described previously; the model for N_2_O had *Spl*(*vDay* + *vDay.Depth* + *vDay.Side*) as the spline term, and the model for CH_4_ only had *Spl*(*vDay*), in this case there being no difference in the curvature over time with respect to depth or side (Supplementary data). In the CO_2_ concentrations, there was a significant interaction between *AirvsHeap*, *Depth* and *vDay* (*F* = 26.55 on 1 and 60 df, *p* < 0.001), showing an effect of depth and time (Fig. 7a); the *AirvsHeap* by *vDay* interaction was also significant (*F* = 22.81 on 1 and 62 df, *p* < 0.001) but the *AirvsHeap* by *Depth* interaction was not (*F* = 1.94 on 1 and 19 df, *p* = 0.180), suggesting that the effect of time was stronger than that of depth. Figure 7b shows the trend recognised by the spline term along with the *AirvsHeap* by *Depth* by *vDay* interaction in Fig. 7a.

The peak in CH_4_ was not as well-defined as in the East Midlands heap; however, there was a significant interaction between *AirvsHeap*, *Depth* and *vDay* (*F* = 8.23 on 1 and 94 df, *p* = 0.005) (Fig. 7c); again, the *AirvsHeap* by *vDay* interaction (*F* = 6.32 on 1 and 94 df, *p* = 0.014) was significant but the *AirvsHeap* by *Depth* interaction was not (*F* = 2.14 on 1 and 17 df, *p* = 0.161). Greater concentrations of N_2_O were detected from the deeper probes, with a significant interaction between *AirvsHeap*, *Depth*, *Side* and *vDay* (*F* = 10.72 on 1 and 46 df, *p* = 0.002) (Fig. 7e) indicating evidence of a different trend over time for the five sample zones, and with the *shape* of trend being modified with respect to both depth and side. Figure 7f shows the effect of depth and side given the spline terms, with the dark side having greater levels of N_2_O than the sunny side. In both instances, the deeper probes detected higher levels of the gas, and there was an overall marginally significant positive relationship of N_2_O with height (*F* = 4.59 on 1 and 25 df; *p* = 0.042; coefficient 0.070, SE 0.040). No effect of height was observed for CO_2_ or CH_4_.

## Discussion

### Dry Matter Losses During Wood Chip Storage

The results from this study suggest that outside wood chip storage is not an efficient method of storing biomass, though this may be location-specific to our study sites, as there are some studies showing lower dry matter losses, and there may be other methods of storage that should be explored (discussed later in this sub-section). In this study, the Rothamsted heap lost around 7 % dry matter per month, compared to the 1 % suggested rule-of-thumb [6]. Reported experience from the literature shows a large range of dry matter losses of between 0.25 and 7.2 % across studies, or between 1 and 27 % over 5 to 13 months of storage [23]; therefore, there is high variability from studies. Differences could be due to a range of factors including the biomass type/species, particle size, moisture content, the season when cut, the age when cut, geographic location and weather, positioning, and the heap size and geometry. A possible reason for the high dry matter losses found in the study is that the bark-to-wood ratio of willow is much higher than in forest-derived wood chips and higher than in poplar. As bark contains many plant nutrients, this may mean that chipped willow SRC is an ideal growth medium for bacteria and fungi [24]. Losses of 1 % per month are consistent with Pari et al. [11] who built a slightly smaller heap of poplar SRC chips in Savigliano, Italy. They observed rapid temperature increases and a loss of 10 % over 7 months. Good weather conditions meant that the chips dried effectively from 70 to 35 %, suggesting that heap storage may be more suited to areas with high seasonal temperatures. Manzone et al. [25] reported losses of 1.6 % dry matter per month in poplar chips in Italy. Wihersaari [19] reported higher losses in forest residue chips, 3.6 % per week for the first 2 weeks of storage, afterwards reducing to 0.4–0.7 % per week. Their study was based in Sweden; therefore, the climatic conditions at the heap may be a determinant of how successful the storage phase will be. The authors suggested that low humidity (under 20 %) is best for reducing losses, which can only be achieved with artificial drying, whereas during the current experiment, humidity averaged 78 and 82 % at Rothamsted and East Midlands, respectively.

It is not clear whether a loss rate ‘per month’ effectively represents *when* losses occur during storage, as suggested by Wihersaari [19]. The experimental design meant that it was not possible to record monthly losses as removing bags during the course of the experiment would have disrupted the crust formation. Also, in biomass supply chains, dry matter losses tend to be caused by microbial activity as well as spillage of material during handling and storage [12]. From the temperature profile and the observed dry matter losses from the two piles, there is a strong indication that the wood chips underwent microbial-derived decomposition [26]. Chemical oxidation of the biomass or physical forces of condensation and adsorption cause much slower development of heat [10], and is usually the dominant heating process when storing low dry-matter biomass feedstocks, such as pellets [27]. The temperature profiles indicate that the wood chips had completed the thermophilic degradation of readily available carbohydrates after 2 months of storage. This may explain why the bagged samples within the two heaps had similar dry matter losses, even though the East Midlands heap was left for longer. Another suggestion would be that the degradation rate at the East Midlands site was slower; however, the temperature and GHG profile do not support this theory.

The initial heating phase is identified as the mesophilic phase in composting [28], and is believed to begin when the material is chipped, either due to a wound response by the still living cells of the SRC [6] or due to the microbial degradation of easily degradable parts of the wood, these mainly being soluble carbohydrates [10]. An immediate rise in temperature was also observed in other studies on SRC chip heaps [29], poplar crown chips [30] and pine chips [10]. As the temperature within the heap increases, temperature-tolerant bacteria dominate the decomposition processes in the thermophilic phase [31]. After the accessible carbon sources have been consumed, the more resistant compounds such as cellulose, hemicellulose and lignin begin a slower process of degradation. This is mainly attributed to the action of woody decay fungi, which require considerable metabolic energy to breakdown the recalcitrant biomass, leading to a drop in both temperature [10] and rate of CO_2_ release [28]. This was observed in both of the wood chip heaps (discussed in the following sub-section).

**Fig. 8:**
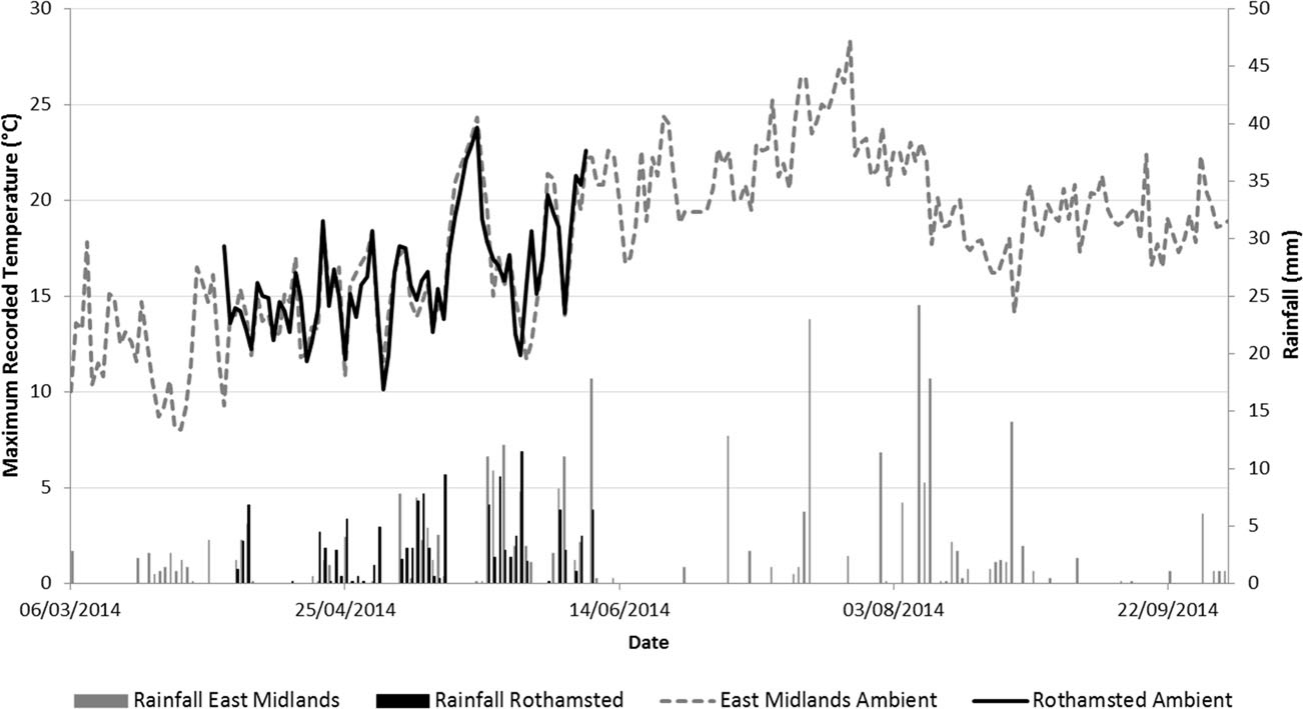
Ambient temperatures and rainfall at the two sites. The East Midlands heap was constructed on 6 March 2014 and the Rothamsted heap was constructed on 3 April 2014

Although the process of self-heating was rapid in both heaps, the Rothamsted heap heated more rapidly than the East Midlands heap, which could have been due to the higher ambient temperatures at the former site, whereas early on at East Midlands there were a few days with a maximum temperature less than 10 °C (Fig. 8). It is possible that the concrete at Rothamsted provided some insulation. It is known that a higher leaf content, as with the Rothamsted heap, would have provided more readily available carbohydrate and nutrients for initial microbial establishment [32], which may have caused this difference in temperature profile.

At East Midlands, the higher C/N ratio indicates that the material was less recalcitrant to breakdown, which could explain why this heap maintained a slightly higher temperature for longer duration compared to Rothamsted, despite similar ambient temperatures and rainfall during this time (Fig. 8). This could also be due to the compaction that occurred during heap construction, which could have hindered the dissipation of heat from the heap [6] and diffusion of air into the heap. The heap was also larger than the one at Rothamsted, potentially contributing to heap self-compaction and insulation of the core region. A study examining moisture content changes in 3 and 6 m high wood chip heaps found that the larger heaps dried more successfully [29].

As expected, a crust layer formed on the outside of the wood chip heaps during the course of the experiment, similar to that reported by Pari et al. [11], who identified a 3-cm outside layer of dry material, then a 20–25-cm wetter layer. In our experiment, the crust appeared to be inundated with white mould which was generally absent on the core regions. It is suggested that high heap temperatures can hinder fungal growth, whereas the optimal temperature for most wood rotting fungi is the mid 30 °C’s, and they are generally thermo-tolerant rather than thermophilic [6]. This may suggest why the mould growth was restricted to the outer layers. The phenomenon was observed by Jirjis [33], who found that ventilating wood chip heaps by placing a tunnel beneath the heap meant that heap temperatures were lower; however, dry matter losses were increased due to the favourable environment for fungal growth. This result was not found in a repeated study in 2005, where the fungal growth rates of comparatively smaller and larger heaps were similar, but dry matter losses were not studied [29]. Noticeable compositional changes occurred in the damper outer layers of the heaps, in regards to nitrogen and carbon content, which may be derived from the fungal material itself.

It is not possible to determine whether losses of dry matter were higher in the crust layer as sampling from the outer layers of the heap, or placing net bags on the outskirts of the heap, may have disturbed crust formation and protection from rainfall. The centre regions of the heap dried quite successfully; however, mixing the entire heap gave a wetter overall chip. Jirjis [29] suggested that variation of the moisture content throughout the heap is a result of the redistribution of moisture within the heap and results in a less homogeneous fuel. The wet region in the top of the heap represents an area of condensation resulting from a ‘chimney effect’ of the air flow within the heap. This has been observed in wood pellet silos [34] and compost windrows [28, 35] and is a result of an unavoidable natural convection process [6]. There may be some scope to avoid this part of the heap during heap breakdown, or the impact of this zone may be reduced by building the largest possible heap, which would also reduce the core-to-crust ratio.

Although it is not possible to compare the two heaps statistically, it has already been suggested that concrete floors can help to reduce losses and chip contamination [36], which is also observed here from obvious additional losses to the ground. Paved areas are suggested to allow less water to come from the ground [11]; however, it is not possible to directly assess this factor as the heaps were left for different durations, were different sized and in different locations. In comparison, a recent study [37] showed virtually zero dry matter losses when storing poplar rods outside in windrows, while drying the material effectively. Manzone et al. [25] found that covering wood chip heaps with plastic or fleece reduced dry matter losses only slightly, and not by enough to justify the cost of covering. Therefore, the issue may be with the harvesting, as wood chips, rather than with how to effectively store the chips. Minimising dry matter losses from a biomass supply chain is important for optimising land use efficiency, therefore other methods of harvesting SRC and hence different forms of the material for storage (e.g. as rods) should be explored. The increased costs of handling uncomminuted biomass would need to be considered, however [17].

In biomass, ash is derived from the minerals that the tree has incorporated during its lifetime and that which originates from contamination during handling [16]. It is believed that the component of wood broken down by microbes will affect the quality of the resulting chip, for example breakdown of the carbohydrate components will lead to an increase in the ash content of biomass [38]. At the Rothamsted site, the ash contents of the biomass were slightly greater after storage compared to the original chip, whereas in contrast at the East Midlands site there was a net decrease in ash. Eisenbies et al. [39] showed that ash concentrations from freshly harvested SRC willow ranged between 0.8 to 3.5 % from a sample of 24 trucks, so it may be difficult to detect changes in ash due to storage. As in both heaps, the relative carbon content of the stored wood chips was unchanged before and after storage, the dry matter losses would have resulted in a loss of carbon from the wood chip, but there was no significant change in ash to support the hypothesis that the carbon losses mainly occurred in the carbohydrate fraction of the biomass.

### Greenhouse Gas Concentrations in Wood Chip Heaps

The GHG concentration profiles detected within the two heaps were significantly different to the ambient samples and showed significant curvature over time. The pattern of results is consistent with those from composting studies [28, 40, 41]. The mesophilic and thermophilic stages of composting are associated with an initial high rate of CO_2_ production that peak as the micro flora move from readily available to more recalcitrant substrates. This is associated with an observed peak and decline in temperature patterns [41] and a loss of dry matter [32]. The initial CO_2_ concentrations detected within the Rothamsted heap were more than double the peak level at East Midlands, which may be attributed to the material at Rothamsted being leafier and hence having a higher soluble carbohydrate fraction, leading to faster degradation rates.

Carbon dioxide was the only GHG present in appreciable quantities in both heaps, as also found by Ferrero et al. [10], indicating that aerobic processes predominated. An effect of side was detected in the East Midlands heap for CO_2_. Higher concentrations were detected on the south facing side, which would be expected to be warmer, driving faster rates of metabolism of associated microorganisms, which is also expected to increase CH_4_ production [18, 42–45]. The effect of side was not significant for CH_4_, however, nor was there a correlation between temperature and CH_4_ concentration.

The profile of CO_2_ concentration was similar in both heaps in that there was a rapid peak and decline. The CH_4_ concentrations differed considerably. In the East Midlands heap, there was a clear upward trend in CH_4_ concentration that occurred as the CO_2_ concentration peaked. The CH_4_ concentration increased over a period of 26 days and then declined over another 21 days. At peak CH_4_ concentration, the CO_2_ to CH_4_ ratio was 40:1, which is much greater than found in an anaerobic digestion system (3:2), suggesting that the environment within the heap is not favourable for methanogens [18]. The mechanism of formation of CO_2_ and CH_4_ (and CO) from woody biomass storage is at present not entirely clear [46]. Methane production is usually attributed to the anaerobic reactions of microorganisms [47] and occurs after the O_2_ has been depleted by aerobic processes, leading to an accumulation of CO_2_, and after bacteria have broken plant material to smaller soluble intermediates [48]. This could explain why the accumulation of CH_4_ in the East Midlands heap occurred after CO_2_ concentration peaked, suggesting that after an active period of aerobic respiration the levels of O_2_ fell. A study on the concentrations of CH_4_ in the headspace of pellet silos, however, concluded that its production was independent of O_2_ levels [43], and a recent report showed negligible CH_4_ production in aerobic and anaerobic forest residue chip containment [32]. The O_2_ concentrations from the two heaps in this experiment were not recorded in this experiment, so its role therefore remains unclear as regards the mechanism of CH_4_ build-up.

Temperature differences between the heaps may explain the difference in CH_4_ concentration patterns, as this can indicate the rate of microbial activity. Also, compaction is known to contribute to CH_4_ production, and it is generally recommended that heaps are not driven over during construction [20]. These factors could be synchronous to larger heaps, such as for East Midlands, as there may have been some self-compaction that allowed temperatures to build up. A smaller heap, as at Rothamsted, may allow more effective ventilation, which avoids development of aerobic conditions in the heap, or otherwise more efficient dispersal of gases.

Methane concentrations were greater towards the core of the heaps. Similar results were reported for sealed pellet silos [47], although in their study the gas stratified over time so that similar concentrations were detected throughout the silo. In the case of the wood chip heap, the gas could therefore be expected to migrate away from the source and eventually leave the heap. Although the method employed in the current study to detect GHG production in the heap can provide some spatial and temporal resolution to the processes of GHG generation within the heap, it suffers from a drawback of not being able to detect the rate at which the gases leave the heap [20]. The majority of studies examining gas emissions from wood pellets during silo storage find that CO_2_ emissions are the greatest and CH_4_ emissions the least. [49] Comparing storage of forest residues with pellets found similar levels of CH_4_ [18].

In biological systems containing CO_2_, O_2_ and CH_4_, it is possible that CH_4_ is oxidised by methanotrophic bacteria to H_2_O and CO_2_ [42]. Some studies have attempted to quantify this by using chambers on the periphery of storage heaps, e.g. Sommer and Moller [41] while studying compost heaps. They found that emissions of CH_4_ did not occur until the concentration at the centre of the heap reached 500 ppm, which can be explained by efficient oxidation of CH_4_ in the surface area of the compost heap. Similarly, a study on sawdust heaps measured concentrations of CH_4_ of between 4 and 63 % within the heap; however, much lower concentrations were measured at the surface using chambers [21]. Hence, there is evidence that the CH_4_ measured within the East Midlands heap, where concentrations reached 1633 ppm, may have led to an emission from the heap. This would compromise the GHG emission savings achievable from utilising woody biomass stored in this way. An improved measurement method is described by Anderson et al. [35], who used a dynamic plume method to observe GHG fluxes from a compost windrow. They concluded that this method best explained the dry matter losses experienced in the heap compared to flux chamber and funnel chamber methods. The dynamic plume method covers the majority of the heap and records the flux of GHG in a similar method to techniques used to measure emissions from land areas, but with a mobile Fourier transform infrared (FTIR) system that monitors gas release every 40 s [35].

Nitrous oxide concentrations were very small in both locations. The relationship between the heap temperature and N_2_O concentration in the East Midlands heap was the opposite to that predicted by Wihersaari [14] and observed in a composting study [50]. The higher peak for the ‘sunnier’ side of the Rothamsted heap suggests there could be an effect of temperature or of moisture availability. Both heaps showed a similar decline in N_2_O concentration after initial establishment of the heap and this may be due to some factor other than temperature.

## Conclusions

The results suggest that heaped outside wood chip storage is not an efficient method of storing willow wood chips because it could lead to dry matter losses in the region of 20 % after 97 days of storage. Although the core of the heaps dried well, the outer layer of chip became wet during storage due to the re-distribution of moisture and from rainfall. As a result, after the piles were moved and mixed, the average moisture content was 10–16 % higher than that found in the core. Due to a combination of the dry matter losses and the relatively low moisture loss, it is estimated that there is a net energy loss from this storage method. Some other studies show lower dry matter losses, which may be due to climatic conditions and the composition of willow, which has a high bark content making it an ideal substrate for microbial decomposition.

The results indicate that the dry matter losses are associated with a rapid temperature increase and increase in CO_2_ concentration over the first 2 months of storage. In the East Midlands heap and to a lesser extent the Rothamsted heap, a peak in CH_4_ concentration was detected after the CO_2_ peak. This suggests that after an active period of aerobic respiration the conditions of the heaps became anaerobic. Further research is required to detect whether there are fugitive emissions of CH_4_ from wood chip heaps as this would compromise the GHG emission savings from utilising SRC willow for heat and power generation. There is evidence that harvesting as whole rods or billets reduces dry matter losses, though the cost trade-off would need to be considered.

## Electronic supplementary material

Below is the link to the electronic supplementary material.

ESM 1 (DOCX 15 kb)
