# Supplementary material for: Dry Matter Losses and Greenhouse Gas Emissions From Outside Storage of Short Rotation Coppice Willow Chip
Source: Bioenergy Res. 2015 Oct 29;9:288–302. doi: 10.1007/s12155-015-9686-y (PMC4913936; doi:10.1007/s12155-015-9686-y)
Supplement: Supplementary file 1 — (DOCX 15 kb) [file 12155_2015_9686_MOESM1_ESM.docx]

Supplementary Data

Statistical assessment of correlation over time for the probes using a power model, and of curvature over time overall and with respect to the treatments: air *vs.* stack, depth and side.

| Heap | GHG Concentration | Within probe correlation over time | Heterogeneity over time points | Evidence of Curvature over time, and with respect to depth and side over time |
| --- | --- | --- | --- | --- |
| Rothamsted Heap | Carbon Dioxide | (χ^2^ = 103.83 on 1 df, p < 0.001) | χ^2^ = 74.46 on 16 df, p < 0.001 | χ^2^ = 44.49 on 1 df, p < 0.001; different according to depth χ^2^ = 78.37 on 1 df, p < 0.001 |
|  | Methane | (χ^2^ = 14.46 on 1 df, p < 0.001) | χ^2^ = 222.72 on 16 df, p < 0.001 | χ^2^ = 36.67 on 1 df, p < 0.001; same regardless of treatment: air *vs.* stack, depth or side (χ^2^-tests, p > 0.05). |
|  | Nitrous Oxide | (χ^2^ = 68.92 on 1 df, p < 0.001) | χ^2^ = 778.56 on 16 df, p < 0.001 | χ^2^ = 13.98 on 1 df, p < 0.001; different according to depth (χ^2^ = 16.07 on 1 df, p < 0.001) and side (χ^2^ = 4.49 on 1 df, p = 0.034). |
| East Midlands Heap | Carbon Dioxide | (χ^2^ = 157.02 on 1 df, p < 0.001) | χ^2^ = 84.37 on 16 df, p < 0.001 | χ^2^ = 28.99 on 1 df, p < 0.001; different according to side (χ^2^ = 3.86 on 1 df, p = 0.049) |
|  | Methane | (χ^2^ = 279.22 on 1 df, p < 0.001) | χ^2^ = 465.21 on 16 df, p < 0.001 | χ^2^ = 4.85 on 1 df, p = 0.028; different according to depth (χ^2^ = 18.14 on 1 df, p < 0.001) |
|  | Nitrous Oxide | (χ^2^ = 116.87 on 1 df, p < 0.001) | χ^2^ = 195.11 on 16 df, p < 0.001 | χ^2^ = 68.92 on 1 df, p < 0.001; different according to depth (χ^2^ = 8.72 on 1 df, p = 0.003) |
